# Botulinum Neurotoxin Serotypes Detected by Electrochemical Impedance Spectroscopy

**DOI:** 10.3390/toxins7051544

**Published:** 2015-05-06

**Authors:** Alison C. Savage, Nicholas Buckley, Jennifer Halliwell, Christopher Gwenin

**Affiliations:** School of Chemistry, Bangor University, Bangor, Gwynedd, Wales LL57 2DG, UK; E-Mails: a.savage@bangor.ac.uk (A.C.S.); chu004@bangor.ac.uk (N.B.); j.h.halliwell@bangor.ac.uk (J.H.)

**Keywords:** electrochemical impedance spectroscopy, biosensor, SNAP-25, VAMP, botulinum neurotoxin

## Abstract

Botulinum neurotoxin is one of the deadliest biological toxins known to mankind and is able to cause the debilitating disease botulism. The rapid detection of the different serotypes of botulinum neurotoxin is essential for both diagnosis of botulism and identifying the presence of toxin in potential cases of terrorism and food contamination. The modes of action of botulinum neurotoxins are well-established in literature and differ for each serotype. The toxins are known to specifically cleave portions of the SNARE proteins SNAP-25 or VAMP; an interaction that can be monitored by electrochemical impedance spectroscopy. This study presents a SNAP-25 and a VAMP biosensors for detecting the activity of five botulinum neurotoxin serotypes (A–E) using electrochemical impedance spectroscopy. The biosensors are able to detect concentrations of toxins as low as 25 fg/mL, in a short time-frame compared with the current standard methods of detection. Both biosensors show greater specificity for their compatible serotypes compared with incompatible serotypes and denatured toxins.

## 1. Introduction

Often, in the treatment of life-threatening illnesses, the speed of diagnosis is paramount to the patient’s recovery and prevention of any long-lasting side effects. This is particularly important in the treatment of botulism, a debilitating disease, which can result in paralysis and death [1].

Botulism is the result of a toxin, botulinum neurotoxin, the deadliest biological toxin known to mankind with an median lethal dose LD50 of 1 ng per kg bodyweight [2]. Whilst botulism is not a common illness, the ability of its toxin to cause such a serious illness has resulted in the toxin being categorised as a potential biological weapon [1,3,4]. Thus, this threat has led to the need for the development of a rapid sensor for the detection of these active toxins for the purpose of biosecurity and testing on suspected cases of botulism. The current method for the detection of all serotypes of botulinum neurotoxins is the mouse lethality assay, which is a time consuming assay involving the use of live animals with a sensitivity of 10 pg/mL of botulinum neurotoxin [5]. Cell-based assays are being offered as an alternative, however, they still take a relatively long time to perform in the order of days [6,7]. Botulinum neurotoxin (BoNT) exists in eight known serotypes, labelled A-H, with the most recently identified serotype being reported in 2014 [8,9]. Its mode of action is targeting and cleaving the soluble N-ethylmaleimide-sensitive attachment protein receptor (SNARE) proteins SNAP-25 and vesicle associated membrane protein (VAMP), prohibiting the release of acetylcholine from nerve terminals resulting in muscle weakness [10]. BoNT/B, BoNT/D, BoNT/F and BoNT/G are known to cleave the VAMP at different amino acid positions, whilst serotypes BoNT/A, BoNT/C and BoNT/E cleave the protein SNAP-25. BoNT/C is also known to target the protein syntaxin [11]. The mode of action of serotype H is yet to be established and verified due to its recent isolation and identification.

A variety of alternative techniques have been reported for the detection of multiple serotypes of botulinum neurotoxin, such as mass spectrometry [12,13], ELISA [14], protein antibody microarrays [14,15], and PCR techniques [16]. Electrochemical techniques are a rapidly growing field of study in the development of biosensors due to its sensitivity and its relative simplicity to run [17]. In particular, advances in this area have led to the development of a miniaturised potentiostat as a convenient electrochemical point-of-care biosensor for cortisol [18]. Electrochemical impedance spectroscopy (EIS) is a sensitive electrochemical technique for probing changes that occur at the surface of an electrode, and has been utilised in the development of sensors for various different disease biomarkers [17,19,20,21]. Compared to electrochemical techniques, such as cyclic voltammetry, EIS is less destructive to protein layers bound to a gold surface due to the comparatively lower potentials used [22]. This allows for direct comparison between the original protein layer and the layer after incubation with the molecules being detected [23].

Herein we report the development of two electrochemical impedance biosensors using the SNARE proteins SNAP-25 and VAMP on gold electrodes for the detection of different serotypes of botulinum neurotoxin. Previously we have reported the SNAP-25 electrochemical impedance biosensor for the detection of active BoNT/A in the pharmaceutical botulinum neurotoxin Dysport^®^, capable of identifying active toxin in the presence of stabilising excipient human serum albumin (HSA) and lactose at concentrations as low as 25 fg/mL [24,25]. Building on from the success of this sensor for the detection of active BoNT/A in the pharmaceutical samples, we have applied it to the detection of native BoNT/A, as well as serotypes BoNT/C and BoNT/E. We have also developed a VAMP sensor for the detection of serotypes BoNT/B and BoNT/D, and demonstrated its ability to detect the presence of these different serotypes in their active form. Table 1 shows the target proteins for the different botulinum neurotoxins used in this study and the amino acid fragment cleaved from the protein by the active BoNT serotypes.

**Table 1:** Botulinum neurotoxin serotypes investigated in this study and their target SNARE protein and amino acid sequence cleaved from the protein upon action of toxin [26,27].

Serotype	Target Protein	Sequence Cleaved from Protein	No. Amino Acids Cleaved
BoNT/A	SNAP-25	RATKMLGSG	9
BoNT/B	VAMP	FETSAAKLKRKYW	13
BoNT/C	SNAP-25	ATKMLGSG	8
BoNT/D	VAMP	LSELDDRADALQAGASQFETSAAKLKRKYW	30
BoNT/E	SNAP-25	IMEKADSNKTRIDEANQRATKMLGSG	26

## 2. Results and Discussion

### 2.1. Formation of SNAP-25 and VAMP Monolayers

**Figure 1:**
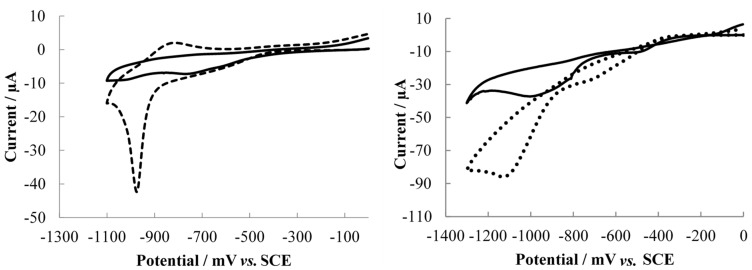
Voltammograms at a sweep rate of 50 mVs^−1^ in 0.1 M NaOH; the working electrodes were SNAP-25 modified Au (111) (─ ─ ─), VAMP modified Au (111) (●●●●) and a bare Au (111) slide (───). SNAP-25 modified Au (111) and bare Au (111) voltammograms reproduced from [25].

The protein monolayers are assembled on the gold electrode surface through the formation of bonds with their naturally occurring sulfurs. This was achieved by incubation of gold electrodes in the protein solution at room temperature for 48 h. Whilst the SNAP-25 contains four cysteines for binding to the gold surface, the VAMP contains methionine residues, which are also able to bind to the gold surface [28,29]. The surface binding of the VAMP protein to the gold electrode was examined by performing stripping voltammetry (Figure 1).

The stripping voltammograms of the SNAP-25 modified gold electrodes show a reduction peak at −970 mV, indicative of the reduction of a gold-thiol bond [30]. The oxidation peak at −850 mV shows the reabsorption of the thiols onto the gold surface.

The VAMP protein showed a shift in the reduction peak to −1100 mV. The two proteins used in this study differ in size, with the SNAP-25 protein comprising of 206 amino acids, which are bound to the gold by sulphurs in the middle of its structure. This conformation gives the SNAP-25 an effective chain length of 103 amino acids. The VAMP protein contains 126 amino acids, this longer chain length is more resistant to ion transport causing the shift to more negative potentials [31].

### 2.2. Electrochemical Impedance Spectroscopy for the Detection of Botulinum Neurotoxin

**Figure 2:**
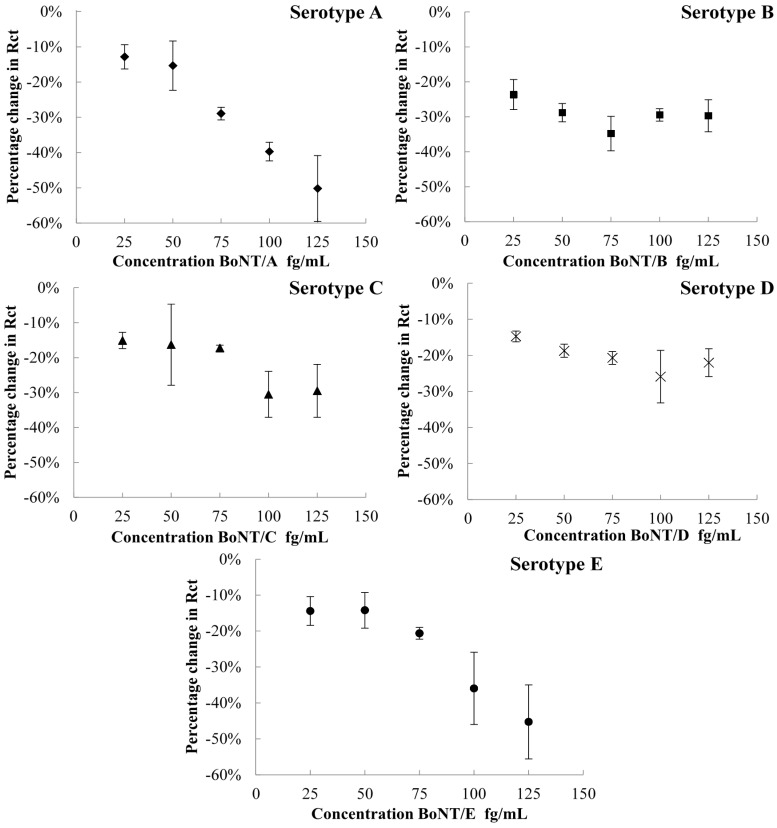
Graphs showing percentage difference of charge transfer resistance against a range of botulinum neurotoxins; serotype **A** (♦) serotype **C** (▲) and serotype **E** (●) using the SNAP-25 modified gold electrode and serotype **B** (■) and serotype **D** (x) using the VAMP modified gold electrode. Errors calculated to ±1 SD.

Electrochemical impedance spectroscopy was employed to measure the impedance of the ferri/ferrocyanide redox probe at the gold working electrode modified with the proteins SNAP-25 and VAMP. The SNAP-25 and VAMP electrodes were used to detect different serotypes of botulinum neurotoxin. After incubation of the electrode with toxin, the charge transfer resistance decreases due to the cleavage of a small fragment of the protein. The assay was performed, in triplicate, with a new electrode for each measurement over a concentration range of 25 fg/mL to 125 fg/mL. Different serotypes were run on their corresponding protein electrode, with serotype A, C, and E measured on SNAP-25 electrode and serotype B and D measured on the VAMP electrode. The calibration graphs for each serotype on the appropriate electrode are shown in Figure 2.

#### 2.2.1. Detection of Botulinum Neurotoxin Serotypes A, C, and E

**Figure 3:**
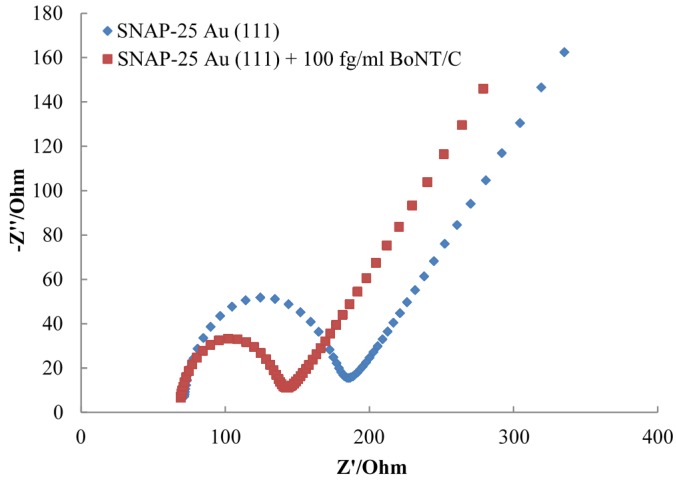
Nyquist plot showing a decrease in charge transfer resistance of SNAP-25 Au (111) after incubation with 100 fg/mL BoNT/C.

The SNAP-25 modified electrodes were used to detect the activity of native botulinum neurotoxins BoNT/A, BoNT/C and BoNT/E. The use of this electrochemical impedance assay with the pharmaceutical Dysport^®^ containing BoNT/A has previously been reported [25]. The pharmaceutical sample contains stabilising excipients HSA and lactose, which could explain the lack of linear response observed using the pharmaceutical sample in the previously published work [25]. A typical Nyquist plot is shown in Figure 3, and the charge transfer resistance was calculated by fitting the data to a Randles circuit. The decrease in charge transfer resistance has been calculated as a percentage of the original SNAP-25 or VAMP layer at different concentrations. This takes into account any differences in the formation of the individual protein layers that may occur.

The typical Nyquist plot of the SNAP-25 biosensor shows a decrease in charge transfer resistance upon incubation with the toxin. The SNAP-25 monolayers were incubated over a range of different concentrations with the three toxins BoNT/A, BoNT/C and BoNT/E. The calibration graphs for BoNT/A, BoNT/C and BoNT/E on SNAP-25 monolayers are shown in Figure 2. Interestingly, the serotypes A and E show similar responses over the same concentration range despite their different modes of action upon the SNAP-25 protein. The three serotypes cleave different size amino acid chains from the SNAP-25 protein (Table 1), with BoNT/E cleaving 26 amino acids, BoNT/A cleaving 9 amino acids and BoNT/C cleaving just 8 amino acids [26]. BoNT/C does not show an obvious linear response when incubated with the SNAP-25 monolayer even though it is reported to cleave just one amino acid different to BoNT/A. This differing response could indicate that BoNT/C does not cleave SNAP-25 very efficiently; however a clear response is still detected. Previous research has suggested that BoNT/C can only cleave SNAP-25 in intact cells due to its conformation and other factors inside the cells [32]. As the SNAP-25 monolayer is bound to the gold surface using the four cysteines used in membrane anchorage its conformation within the cells in mimicked in the sensor, however other molecules usually found within cells are not present which could explain the lower activity at higher concentrations of toxin. Incubation of the SNAP-25 electrode with serotypes A and E shows a linear decrease in charge transfer resistance upon increasing concentrations. This suggest that increasing concentrations causes an increase in proteolytic activity on the gold electrode surface, thus decreasing the impedance of oxidation and reduction experienced by the redox probe at the gold surface compared to the original SNAP-25 monolayer.

#### 2.2.2. Detection of Botulinum Neurotoxin Serotypes B and D

**Figure 4:**
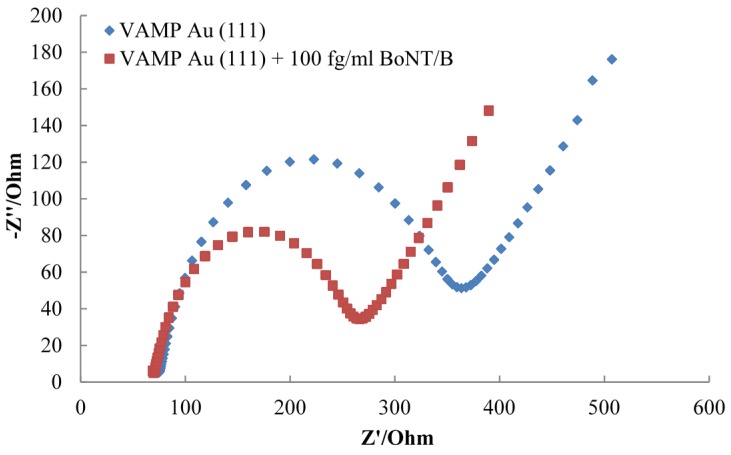
Nyquist plot showing a decrease in charge transfer resistance of VAMP Au (111) after incubation with 100 fg/mL BoNT/B.

Serotypes BoNT/B and BoNT/D have been detected by EIS using VAMP coated gold electrodes. A similar decrease in charge transfer resistance occurs upon incubation of the protein layer with the toxin. A typical Nyquist plot for the VAMP monolayer before and after incubation with BoNT/B is shown in Figure 4. A decrease in charge transfer resistance compared to the original VAMP monolayer has been observed with all concentrations of BoNT/B and BoNT/D, over the same concentration range carried out with serotypes BoNT/A, BoNT/C and BoNT/E.

Both serotypes show a decrease in the percentage change in charge transfer resistance as the concentration of toxin increases. As with the previous serotypes, the two toxins have different modes of action, with the BoNT/B cleaving a 13 amino acid chain from the VAMP protein and the BoNT/D cleaving a longer 30 amino acid chain [27]. Compared to serotypes A and E with the SNAP-25 biosensor, the VAMP electrode shows a smaller percentage change in the charge transfer resistance across the concentration range for both serotypes. The response from the serotypes tested with VAMP also showed a plateau at higher toxin concentrations compared to BoNT/A and BoNT/E, which continued in their linear response. This indicates the toxin has cleaved all of the available protein so the charge transfer resistance cannot decrease further. The plateau and smaller response could be due to the way in which the protein is packed onto the gold surface. If the site at which the protein is cleaved is not easily accessible by the toxin it will affect the proteolytic activity. The lower potentials measured in the stripping voltammetry for the VAMP electrode (Figure 1) suggests that the VAMP protein may be packed better on the surface of the electrode compared with the SNAP-25 protein, potentially reducing the accessibility of the cleavage site. The SNAP-25 protein is anchored to the gold surface by four cysteines, which are also responsible for anchoring the protein to the neural plasma membrane. Thus the protein should sit on the gold surface in the same conformation as found *in vivo*, making the cleavage sites freely accessible to the toxin. The structural conformation of the VAMP protein on the gold surface is not known, but it is possible that it may have adopted a different conformation to that found on membranes *in vivo*.

### 2.3. Cross Reactivity of Different Serotypes

**Figure 5:**
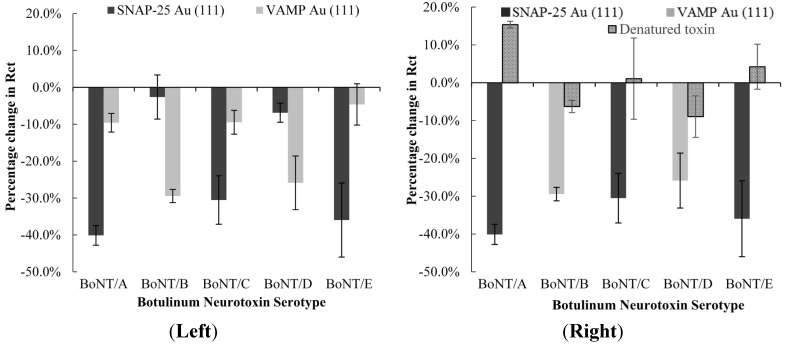
(**Left**) Reactivity of the different BoNT serotypes on the SNAP-25 and VAMP modified electrode and (**Right**) Reactivity of active and denatured toxin on SNAP25 and VAMP electrodes. The electrodes were incubated with 100 fg/mL toxin, with errors were calculated to ±1 SD.

In order to determine the specificity of each of the biosensors for their respective serotypes, each of the different serotypes were tested on the SNAP-25 or VAMP electrodes at the same concentration. The results show a much larger percentage change in charge transfer resistance upon incubation with active compatible toxins that are known to cleave the proteins (Figure 5). This provides evidence of the specificity in the mode of action of each toxin serotype.

Toxins were denatured by heating for 15 min at 95 °C, and tested on their respective electrodes (Figure 5). Incubation of denatured BoNT/A, BoNT/C and BoNT/E with the SNAP-25 electrode showed an increase in charge transfer resistance for all three serotypes, consistent with results previously observed [25]. Denatured BoNT/B and BoNT/D on VAMP electrode shows a small decrease in charge transfer resistance, with the same concentration of active toxin giving a much greater response. The VAMP electrode shows a similar response upon incubation with active incompatible toxin and denatured compatible toxin, which suggests that this is a response of the VAMP modified electrode due to repeated washings and incubation in a solution at 37 °C, and the larger responses observed with the compatible toxins due to the presence of the active form of the toxin.

The overall response of the incompatible toxin serotypes and the denatured toxins is relatively small (largest negative response is approximately −10%) compared with the responses observed for the active toxins on their compatible electrodes (between −25% to −40% dependant on serotype). Whilst it may not be possible to determine the exact serotype acting upon the electrode due to the similar response observed for different serotypes, it is possible to determine the presence of active toxins of all serotypes and narrow down a shortlist of potential candidates depending on which electrode it gives the strongest response. As each of the serotypes are pre-treated using the same procedure, this could allow for the development of a neural network dependant on responses observed on each biosensor to determine the serotype.

## 3. Experimental Section

Gold working electrodes were purchased from Winkler GmbH Germany. SNAP-25 was purchased from Abcam, VAMP from Antibodies Online GmbH. Sodium hydroxide (99.99%), potassium chloride (99.999%), potassium ferri/ferrocyanide, zinc chloride, dithiothreitol, tween-20 and 4-(2-hydroxyethyl)-1-piperazineethanesulfonic acid (HEPES) were purchased from Sigma Aldrich. All botulinum neurotoxin serotypes were purchased from Miprolab GmbH in their complex form. Toxin samples were prepared as previously described [33]. Water was purified and had a nominal resistivity of 18 MΩ cm at 25 °C.

### 3.1. Self-Assembled Monolayers of SNAP-25 and VAMP

Gold working electrodes were flame annealed to produce the regular Au (111) terraces [34]. After cooling they were placed in a solutions of SNAP-25 (125 µg/mL) or VAMP (125 µg/mL) in water and left to incubate for 48 h at 20 °C.

### 3.2. Cyclic Voltammetry

All measurements were performed using an Autolab PGSTAT 30 computer-controlled electrochemical measurement system (Eco Chemie, Holland) with a home-made three electrode cell with a SNAP-25 or VAMP modified Au (111) working electrode, a platinum counter electrode and a saturated calomel electrode as the reference electrode.

Stripping voltammetry was performed to analyse the binding of the proteins to the gold surface as previously described [25]. Briefly, the electrode was removed from the protein solution, rinsed thoroughly with ultra-pure water and dried under nitrogen before being sealed in the electrochemical cell. NaOH (100 mM, 15 mL) was added and degassed for 20 min with nitrogen. Two cyclic voltammograms were measured between 0 and −1300 mV for the VAMP monolayers and between 0 and −1100 mV for the SNAP-25 monolayers at a scan rate of 50 mVs^−1^.

### 3.3. Electrochemical Impedance Spectroscopy

**Figure 6:**
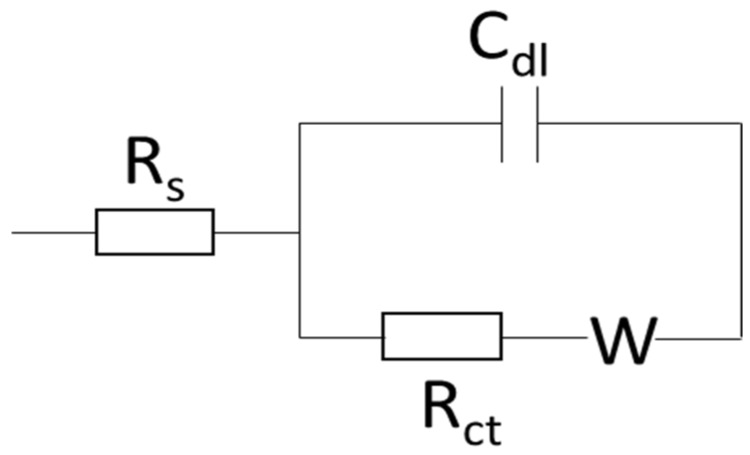
Schematic of the Randles circuit showing the electrolyte resistance (R_s_), charge transfer resistance (R_ct_), double layer capacitance (C_dl_) and the Warburg element (W).

Electrochemical impedance spectroscopy was performed as previously described using ferri/ferrocyanide (5 mM) as a redox probe in potassium chloride (100 mM) at 230 mV with a perturbation amplitude of 10 mVs^−1^ between the frequencies of 25 kHz and 100 mHz and fitted to a Randles circuit (Figure 6) [35].

The initial protein monolayer was probed by electrochemical impedance spectroscopy before the electrode was rinsed with ultra-pure water and incubated with toxin (200 µL in water) for 30 min at 37 °C. The toxin was then removed and electrode washed again with ultra-pure water before the impedance was re-run.

## 4. Conclusions

The electrochemical impedance biosensors offer a quick response to show the presence of active toxin, with results being obtained in a matter of a couple of hours rather than days as encountered with the current established methods. They are able to detect active toxin at concentrations as low as 25 fg/mL, depending on serotype, which is lower than the mouse lethality assay. Comparison with incompatible toxins and denatured toxin control solutions indicate that the large responses observed are caused by the presence of the active toxin rather than any other processes occurring at the surface, and demonstrated the selectivity of the two different biosensors for the different serotypes.

Due to the proteolytic nature of the toxin each electrode can only be used once. Slight variations between SNAP-25 monolayers did not affect the concentration dependant response as the impedance of the monolayer is measured first then repeated after incubation with the toxin and the percentage change in charge transfer resistance used.

The biosensor can be further developed to take into account sensing of the serotypes F and G, which also cleave the protein VAMP. These were not included in this study due to limitations in being able to source these serotypes. Currently this sensor has only been tested in a laboratory environment using buffers as the sample matrices. To test other sample matrices such as body fluids or food simple purification steps would need to be included, such as immunochromotography and centrifugation, to remove non-specific proteases, which may be present. In the future, with the development of miniaturised electrochemical systems, it should be possible to develop these biosensors into a point of care device for diagnostic and detection purposes.
